# Micronutrient Status in Female University Students: Iron, Zinc, Copper, Selenium, Vitamin B_12_ and Folate

**DOI:** 10.3390/nu6115103

**Published:** 2014-11-13

**Authors:** Flavia Fayet-Moore, Peter Petocz, Samir Samman

**Affiliations:** 1 Discipline of Nutrition & Metabolism, School of Molecular Bioscience, The University of Sydney, NSW 2006, Australia; E-Mail: flavia@nraus.com; 2 Department of Statistics, Macquarie University, Ryde, NSW 2112, Australia; E-Mail: peter.petocz@mq.edu.au

**Keywords:** iron, zinc, copper, selenium, vitamin B_12_, folate, biomarker, women

## Abstract

Young women are at an increased risk of micronutrient deficiencies, particularly due to higher micronutrient requirements during childbearing years and multiple food group avoidances. The objective of this study was to investigate biomarkers of particular micronutrients in apparently healthy young women. Female students (*n* = 308; age range 18–35 year; Body Mass Index 21.5 ± 2.8 kg/m^2^; mean ± SD) were recruited to participate in a cross-sectional study. Blood samples were obtained from participants in the fasted state and analysed for biomarkers of iron status, vitamin B_12_, folate, homocysteine, selenium, zinc, and copper. The results show iron deficiency anaemia, unspecified anaemia, and hypoferritinemia in 3%, 7% and 33.9% of participants, respectively. Low vitamin B_12_ concentrations (<120 pmol/L) were found in 11.3% of participants, while 4.7% showed sub-clinical deficiency based on serum methylmalonic acid concentrations >0.34 μmol/L. Folate concentrations below the reference range were observed in 1.7% (serum) or 1% (erythrocytes) of participants, and 99.7% of the participant had erythrocyte-folate concentrations >300 nmol/L. Serum zinc concentrations <10.7 μmol/L were observed in 2% of participants. Serum copper and selenium concentrations were below the reference range in 23% and 11% of participants, respectively. Micronutrient deficiencies including iron and vitamin B_12_, and apparent excess of folate are present in educated Australian female students of childbearing age, including those studying nutrition. The effects of dietary behaviours and food choices on markers of micronutrient status require further investigation.

## 1. Introduction

Young females are at risk of nutrient deficiencies due to poor diets and higher requirements for micronutrients, such as iron and folate especially in the periconceptional period and during pregnancy [1]. Some young women’s dietary patterns which include the avoidance of micronutrient-rich meat and poultry [2], and social influences such as the desire to have a lower BMI despite having a BMI in the normal range [3] resulting in chronic dieting, could further negatively impact their nutrient status and related health outcomes. Multiple micronutrient deficiencies is a worldwide problem, and extends to a range of population groups including young and apparently healthy women [4]. Due to the significant role that vitamin and mineral statuses play in disease prevention, it is important to identify deficiencies in sub-populations. Data on the micronutrient status of women are less readily available and incomplete.

The adequate intake of vitamins and minerals such as iron, zinc, copper, selenium, vitamin B_12_ and folate is essential for optimal health [4]. Iron deficiency (ID) and iron deficiency anaemia (IDA) are common worldwide especially among young women, and have been shown to decrease general health and wellbeing [5,6]. IDA affects work and exercise performance, increases fatigue, and compromises immunity and neurological function [5,6]. IDA has been linked to impaired cognitive function and poor pregnancy outcomes [7] including low birth weight [8].

Both iron and zinc are readily bioavailable from animal products [6,9] and naturally coexist in foods, so marginal deficiencies of both minerals have been associated [6,9]. Zinc, copper and selenium are involved in various aspects of metabolism [4]. Zinc functions in pathways that are necessary for growth, immunity, reproductive function and neuro-behavioural development [9]. Measurement of serum or plasma zinc concentration is currently the only biochemical indicator recommended for the assessment of the zinc status of populations [10]. Copper is a component of many oxidative enzymes, and impacts on iron status, with both high and low copper status being associated with iron metabolism and anaemia [11]. Assessment of copper status has challenges like that of other minerals, and serum copper is used widely as a biomarker of copper status. Selenium plays a key role as an antioxidant through its association with glutathione peroxidase, and has broader functions as a component of selenoproteins [12]. Dietary intake resulting in suboptimal selenium status has been associated with increased risk of cancer, cardiovascular disease, thyroid disease, infertility and adverse reproductive outcomes [13]. There are no recent reports of blood selenium concentrations among young women in Australia.

Vitamin B_12_ and folate are involved in single-carbon transfer and DNA synthesis [4], and are particularly important for young women. Long-term, vitamin B_12_ deficiency impairs cognitive function and it is important in the prevention of neural tube defects (NTD) [4,14]. Vitamin B_12_ is a cofactor in the metabolic transformation of homocysteine to methionine, a reaction that also requires folate [14]. Vitamin B_12_ deficiency has been linked to megaloblastic and pernicious anaemia [4,14] and a decline in cognitive function in older age [15]. Serum vitamin B_12_ is a biomarker of vitamin B_12_ deficiency, and the metabolites, methylmalonic acid (MMA) and homocysteine are functional indicators [4,14]. Elevated plasma total homocysteine (tHcy) has been shown to increase the rate of pre-eclampsia and is related to other poor pregnancy outcomes [16]. Chronic folate deficiency can result in anaemia, and together with vitamin B_12_ deficiency has been associated with cognitive dysfunction, decline in physical function and osteoporosis in the elderly [17]. Vitamin B_12_ is found only in animal products, and therefore predisposes vegetarians, a common dietary practice among young women, to a greater risk of deficiency [14]. High dietary intakes or supplements of folate and vitamin B_12_ decrease elevated plasma tHcy concentrations [18] and reduce the risk of NTD [19,20].

Recognition of nutrient deficiencies in women of reproductive age is important not only because nutritional status affects women’s health and wellbeing, but also because deficiencies are associated with adverse pregnancy outcomes. Also, deficiencies in micronutrients such as folate, vitamin B_12_, iron and trace elements can have adverse consequences on infant mortality and morbidity. There are limited biochemical data on the micronutrient status of young women in Australia. We have undertaken a cross-sectional study in which we collected dietary data, anthropometric parameters and behavioural characteristics of young female university students [2,3]. The aims of the present study are to determine (i) the concentrations of selected micronutrients in women of childbearing age; (ii) the prevalence of deficiency; and (iii) the relationships between micronutrient biomarkers that may exist in this population.

## 2. Subjects and Methods

### 2.1. Subjects

Female students, age 18–35 year participated in a cross-sectional study. They were recruited from the University of Sydney campus using flyers, word-of-mouth referrals, online advertisements, and university publications. Women who reported a diagnosed eating disorder, currently pregnant or breastfeeding, elite athletes, and those not enrolled as students were excluded. A further exclusion criterion was the reported use of nutritional supplements (30% of population reported use) and prescription medication, except for oral contraceptives. Approval to conduct the study was granted by the University of Sydney Human Ethics Committee.

### 2.2. Collection of Blood Samples

Blood samples were collected from an antecubital vein. Subjects were in a fasted state (10–12 h) and in the supine position during the blood collection which took place between 0730 and 0930 h. Blood samples were collected into vacutainer tubes (Becton Dickinson, Franklin Lakes, NJ, USA): EDTA-coated tubes for the subsequent analysis of hemoglobin (Hb), erythrocyte folate (E-folate), tHcy and MMA, and untreated tubes for the analysis of zinc, copper, selenium and iron biomarkers (serum iron, ferritin, transferrin, serum transferrin receptor [sTfR]), serum folate and vitamin B_12_ concentrations. All blood samples were kept on ice for up to 2 h, and centrifuged at 1500 g for 10 min at 5 °C. Serum and plasma samples were obtained and immediately stored at −80 °C until subsequent analysis.

### 2.3. Biochemical Analyses

The biochemical analyses were undertaken in accredited diagnostics laboratories (Royal Prince Alfred and Westmead Hospitals, Sydney, Australia). The concentrations of vitamin B_12_, serum folate, E-folate, ferritin and sTfR were determined using an automated system (UniCel DxI Immunoassay System, Beckman Coulter Inc., Brea, CA, USA). Hb was measured using the Sysmex instrument (Hoffman-La Roche Inc., Nutley, NJ, USA). Serum iron concentrations were determined by a colorimetric method (Cobas Fara II, Hoffman-La Roche Inc., Nutley, NJ, USA). Transferrin concentrations were determined by an automated method (Immage, Beckman Coulter Inc., Brea, CA, USA) and transferrin saturation was determined by dividing the serum iron concentration by the concentration of transferrin, and expressed as a percentage. Plasma tHcy was analysed by HPLC (Homocysteine HPLC kit, BioRad, Munich, Germany) with fluorescence detection (Waters, Milford, MA, USA). Plasma MMA was assayed as described previously [21,22]. Serum zinc, copper and selenium concentrations were determined using inductively coupled plasma mass spectrometry (ICPMS, Agilent 7500ce, Santa Clara, CA, USA) after dilution in an ammonium EDTA-based diluent. Laboratory quality assurance included the analysis of serum from standard pools and international standards. In some cases, due to inadequate plasma volumes for analyses, not all biomarker measurements were completed for all subjects.

### 2.4. Micronutrient Cut Points

Reference ranges are shown in Table 1. The reference range for E-folate of ≥300 nmol/L was applied. A further cut-off ≥906 nmol/L was used since this concentration is associated with a lower risk in NTD-affected pregnancies [23]. There is no universal cut-off for vitamin B_12_ deficiency and levels vary across studies. Therefore, low serum vitamin B_12_ was defined as serum vitamin B_12_ less than the lowest cut-point in the reference range, 120 pmol/L. In addition, biomarkers of folate and vitamin B_12_ were stratified by quartiles of serum vitamin B_12_: <110.7, 111–177, 177.1–221, and >258.2 pmol/L. With respect to selenium, 1.14 μmol/L was chosen as Thomson *et al.*, reported that this level is required to achieve maximal activity of glutathione peroxidase [12]. With respect to zinc, the International Zinc Nutrition Collaborative Group has proposed a concentration of 10.7 μmol/L as the lower cut-off for serum zinc concentrations for females [10]. Regarding iron status, there are several transitional phases from normal iron status to the development of IDA [24]. The three established states in the continuum of iron deficiency are; (i) depletion of iron stores which is reflected by a decrease in serum ferritin; (ii) depletion of functional iron compounds, or iron-deficient erythropoiesis, is reflected by an increase in transferrin, decrease in transferrin saturation or an increase in sTfR; and (iii) IDA is characterised by a decrease in Hb, along with low ferritin and elevated sTfR. A limitation of serum ferritin is that it is elevated in response to infection and/or low-grade inflammation [25]. WHO identifies iron deficiency when the population prevalence for low serum ferritin and elevated sTfR are ≥20% and ≥10%, respectively; and anaemia when Hb < 120 g/L [26]. The Australian Iron Status Advisory Panel has three categories of iron status: iron depletion (serum ferritin <12 μg/L), ID (iron depletion and transferrin saturation <16%) and IDA (ID and Hb <120 g/L) [27]. In the present analysis, we combined the serum ferritin cut points into four categories: <12, 12–15, >15–20, >20 μg/L. Hypoferritinemia was defined as a ferritin concentration <15 μg/L which is the lower limit of the reference range used by the Royal Prince Alfred Hospital laboratory.

### 2.5. Statistical Analyses

Data analyses were performed using SPSS. Pearson’s correlation was used for normally distributed variables and Spearman’s rank correlation coefficient was used to measure associations in non-normally distributed variables. Differences between sub-groups were determined by analysis of variance for continuous variables or chi-square tests for categorical variables. Statistical significance was accepted at *p* < 0.05 for all tests.

## 3. Results

### 3.1. Participants and Baseline Characteristics

A total of 308 women participated in the study. The participants’ mean age was 22.6 ± 3.9 year (mean ± SD), with 81% aged between 18 and 25 year. Mean BMI (21.5 ± 2.8 kg/m^2^) was within the healthy weight range, 78% were in the healthy weight range, 11.0% were underweight, 10.0% were overweight and 1% were obese. There were more students from the Sciences and Health Sciences disciplines than other faculties combined and 20.1% (*n* = 62) were nutrition students (undergraduate or graduate).

### 3.2. Status of Iron, Zinc Copper and Selenium

The participants’ biomarker status of folate, vitamin B_12_, iron, zinc, copper and selenium are shown in Table 1. Low iron status was prevalent in a significant proportion of participants. Relative to the respective reference range, 33.9% of women had low serum ferritin concentrations (hypoferritinemia), 24.6% had elevated serum transferrin concentrations, and 16.6% had low transferrin saturation. IDA, defined as serum ferritin <15 µg/L and Hb < 120 g/L, and unspecified anaemia, defined as Hb < 120 g/L, were seen in 3% and 7% of participants, respectively. When iron biomarker concentrations were stratified by Hb level, the prevalence of hypoferritinemia (<15 μg/L) and transferrin saturation (<15%) increased (*X*^2^, *p* < 0.01) in those with Hb < 120 g/L as compared to those with Hb ≥ 120 g/L. Similarly, as serum ferritin concentrations decreased, all measured biomarkers of iron status and serum folate concentrations were decreased significantly (*p* < 0.001) (Table 2).

Bivariate analyses showed that transferrin saturation was inversely related to serum copper concentrations (*r =* −0.229, *p* < 0.001), and weak, but significant correlations were found between sTfR and serum zinc (*r* = −0.134, *p* < 0.05) and serum copper (*r =* 0.175, *p* < 0.01) concentrations. The mean serum zinc concentration was within the normal range (Table 1), and concentrations <10.7 μmol/L were observed in 2% of participants. Serum copper and selenium concentrations were below the reference range in 23% and 11% of participants, respectively. Women who had serum selenium concentrations >1.14 μmol/L (33%) also had higher serum zinc concentrations (13.3 ± 2.1 *vs.* 14.6 ± 3.0 μmol/L, *p* < 0.0001) and higher serum vitamin B_12_ concentrations (205.9 ± 88.6 *vs.* 800.9 ± 280.5 pmol/L *p* < 0.0001) than women with serum selenium concentrations <1.14 μmol/L.

**Table 1:** Biomarkers of iron, zinc, selenium, copper, vitamin B_12_ and folate statuses in young women.

Biomarker	*n*	Mean ± SD	Median ± SE	Reference Range ^1^	*n* (% above Reference Range)	*n* (% below Reference Range)
Serum iron (μmol/L)	301	17.7 ± 6.8	17.0 ± 0.4	10–30	14 (4.7)	36 (12.0)
Serum transferrin (g/L)	301	2.8 ± 0.6	2.8 ± 0.1	1.8–3.2	74 (24.6)	16 (5.3)
Transferrin saturation (%)	301	25.9 ± 10.9	24.0 ± 0.7	15–50	4 (1.3)	50 (16.6)
Serum ferritin ^2^ (μg/L)	301	28.5 ± 23.7	22.0 ± 1.5	15–150	1 (0.3)	102 (33.9)
Soluble transferrin receptor ^2^ (mg/L)	292	1.51 ± 0.85	1.32 ± 0.05	0.90–2.30	29 (9.9)	33 (11.3)
Haemoglobin ^2^ (g/L)	294	131.2 ± 13.2	132.0 ± 0.7	120–150	7 (2.4)	32 (10.9)
Serum zinc (μmol/L)	289	13.7 ± 2.5	14.0 ± 0.2	10–18	6 (2.1)	6 (2.1)
Serum copper ^2^ (μmol/L)	289	16.0 ± 5.9	13.9 ± 0.4	12.0–22.0	48 (16.6)	66 (22.8)
Serum selenium (μmol/L)	289	1.11 ± 0.21	1.08 ± 0.13	0.75–1.35	2 (0.69)	32 (11.1)
Serum vitamin B_12_^2^ (pmol/L)	302	224.4 ± 109.2	204.0 ± 6.7	120–600	3 (1.0)	34 (11.3)
MMA ^2,3^ (μmol/L)	300	0.17 ± 0.09	0.15 ± 0.01	0.06–0.34	14 (4.7)	0 (0.0)
Serum folate (nmol/L)	302	23.5 ± 10.4	23.0 ± 0.6	7.0–25.0	129 (42.7)	5 (1.7)
E-folate ^2,4^ (nmol/L)	300	839 ± 297	775 ± 19	≥300	299 (99.7)	1 (0.33)
Serum tHcy ^5^ (μmol/L)	307	7.2 ± 2.4	7.0 ± 0.2	5–18	1 (0.33)	26 (8.5)

^1^ Reference ranges as defined by the Royal Prince Alfred and Westmead Hospitals diagnostics laboratories, Sydney, Australia; ^2^ Non-normally distributed; ^3^ Methylmalonic acid; ^4^ Erythrocyte-folate; ^5^ Total homocysteine.

**Table 2:** Concentrations of iron and folate biomarkers stratified by serum ferritin concentrations ^1^.

Biomarkers	Serum Ferritin Concentration (μg/L)			
	<12	12–15	>15–20	>20
Serum iron (μmol/L)	13.8 ± 6.0	16.3 ± 6.9	18.7 ± 6.5	19.3 ± 6.7 *
Serum Transferrin (g/L)	3.1 ± 0.8	2.9 ± 0.7	2.9 ± 0.7	2.7 ± 0.5 *
Transferrin Saturation (%)	18.8 ± 8.9	22.3 ± 8.4	26.2 ± 8.7	29.1 ± 25.5 *
Serum Ferritin (μg/L)	7.3 ± 2.6	13.4 ± 1.2	17.6 ± 1.1	45.0 ± 25.4 *
Soluble transferrin receptor (mg/L)	1.87 ± 0.81	1.27 ± 0.45	1.45 ± 1.25	1.37 ± 0.72 *
Haemoglobin (g/L)	127.3 ± 16.2	130.4 ± 12.1	132.9 ± 8.3	133.5 ± 12.1 *
Serum folate (nmol/L)	20.0 ± 10.0	24.8 ± 10.5	23.5 ± 10.5	24.9 ± 10.2 *
E-folate (nmol/L)	773 ± 276	828 ± 280	831 ± 342	851 ± 300

^1^ Data shown as mean ± SD; * *p* < 0.001 for trend ferritin concentrations.

### 3.3. Status of Folate and Vitamin B_12_

Vitamin B_12_ concentrations <120 pmol/L were found in 11.3% of participants, while 4.7% showed sub-clinical deficiency based on MMA concentrations (>0.34 μmol/L) (Table 3). Concentrations of folate below the reference range were observed in 1.7% (serum) or 1% (erythrocytes) of participants. All (99.7%) but one participant had values above the reference range for E-folate (≥300 nmol/L). E-folate concentrations >500 nmol/L were found in 88.3% of females and a quarter of participants had levels >1000 nmol/L. When stratified according to the cut-off of 906 nmol/L [23], we found significantly lower tHcy concentrations in those above the cut-off as compared to those with E-folate < 906 nmol/L, but no difference in vitamin B_12_ status. Plasma MMA and tHcy concentrations were significantly higher, and serum folate significantly lower as serum vitamin B_12_ concentrations decreased (*p* < 0.01) (Table 3). Significant correlations were found between biomarkers of folate and vitamin B_12_ status. Serum folate concentrations were positively correlated to E-folate (*r =* 0.637, *p* < 0.001) and serum vitamin B_12_ concentrations (*r =* 0.215, *p* < 0.001), and inversely correlated to tHcy (*r =* −0.287, *p* < 0.001). As serum vitamin B_12_ concentrations increased, tHcy (*r =* −0.314, *p* < 0.001) and MMA decreased (*r =* −0.277, *p* < 0.001) significantly. Plasma MMA decreased also as plasma tHcy decreased, although not as strongly correlated (*r =* 0.136, *p* < 0.05).

**Table 3:** Biomarkers of folate and vitamin B_12_ stratified by serum vitamin B_12_ concentrations ^1^.

Serum Vitamin B_12_ Concentrations ^2^ (pmol/L)	*n*	Serum Folate (nmol/L)	tHcy (μmol/L)	E-Folate (nmol/L)	MMA (μmol/L)
<110.7	28	16.6 ± 10.4 *	9.0 ± 2.7 *	781.5 ± 255.7 *	0.21 ± 0.08 *
111–177	87	22.9 ± 10.3	7.7 ± 2.6	789.0 ± 291.9	0.18 ± 0.10
177.1–221	61	22.2 ± 9.4	7.0 ± 1.9	854.8 ± 269.6	0.17 ± 0.09
>258.2	87	25.9 ± 10.3	6.6 ± 1.9	831.5 ± 318.3	0.15 ± 0.07

^1^ Data shown as mean ± SD; ^2^ Quartiles of serum vitamin B_12_; * *p* < 0.001 for trend.

## 4. Discussion

The present study in a group of young women describes the nutritional status of selected micronutrients by using a range of biomarkers. Selection of an apparently healthy group allowed for the assessment of nutrient status and examination of relationships between micronutrients independently of any significant health confounders. The focus of the study was the determination of micronutrient status in young female university students of childbearing age since this group is at risk of deficiency because of the higher micronutrient requirements for maintenance of metabolic stores, and increased requirement potentially due to pregnancy and lactation. The results from the present study show a high prevalence of poor iron status with the most common abnormality being hypoferritinaemia. Other significant findings were the presence of low vitamin B_12_ status concurrent with a dominance of elevated E-folate concentrations.

Hypoferritinaemia and IDA were observed in 33.9% and 3% of participants, respectively. Sub-optimal vitamin B_12_ status, evidenced by low serum vitamin B_12_ and elevated MMA concentrations, were found in 11% and 5% of women, respectively. Optimal selenium concentrations were achieved by a third of participants. In contrast, the participants were not at risk of low folate status, as 99.7% had E-folate concentrations above the reference range.

Serum ferritin concentrations <30 μg/L indicate an increased risk of ID, particularly if iron bioavailability is decreased or iron demands are increased [28]. In the present study, the median serum ferritin concentration (22.0 ± 1.5 μg/L) was similar to concentrations that are reported for other young women [29,30,31] and represent a substantial increase in the risk of adverse functional effects. We found significant relationships between serum ferritin concentrations and all measured biomarkers of iron status, and we confirmed the previous finding that STfR is inversely correlated to transferrin saturation, Hb and serum iron concentration [32].

The mean serum zinc concentration reported in the present study is consistent with other reports, including levels in young women of childbearing age [33], a study in a general French adult population [34], and concentrations were higher than those reported in omnivores and vegetarians [31,35]. Factors that may decrease plasma zinc include vegetarian diets, where as much as 35% of a reduction in plasma zinc is reported [36]. Despite the association between zinc and iron, only a weak negative correlation was seen between serum zinc concentrations and STfR but not with other biomarkers of iron status.

There is limited information on selenium and copper status in Australia. Serum copper concentrations in our study were similar to those observed in women who followed a lacto-vegetarian diet and to that observed in Italian adults [37]. In contrast, our results were slightly lower than non-vegetarians [38], and healthy female adults [39]. In the present study serum copper concentrations below the reference range were observed in 22% of participants. Serum copper concentrations were positively related to sTfR and inversely related to serum ferritin concentrations, indicating an inverse relationship between copper and iron status. The available data for selenium include a mean estimate for plasma selenium concentrations of 1.12 μmol/L and values ranging from 0.97 to 1.54 μmol/L, in relatively small studies [40]. Selenium concentrations in the present study were similar to several studies in healthy adults [34,41,42], including New Zealand and Australian women [12], but were lower than levels in many selenium-sufficient countries including Canada, Sweden, UK and USA [42]. Thomson *et al.*, reported that a plasma selenium concentration of 1.14 μmol/L is required to achieve maximal glutathione peroxidase activity [12]; in the present study 33% of participants reached this target concentration. Further assessment of selenium status among this population may be beneficial in order to promote optimal selenium status.

Serum folate concentrations in Australian females aged 14–45 year (the target group for supplementation) have increased from 14.0 nmol/L, in 1993–1996, to 16.7 nmol/L in 2000; and in 2006, levels were 24 nmol/L. The percentage of low folate concentrations decreased from 8.5% to 4.1% [43], and the present study results showed that only 1.7% have low folate status. The fortification programme in the USA has increased serum and E-folate concentration to levels that are double the projected value [44] and virtually eliminated folate deficiency [45]. Over a third of our study participants had serum folate levels associated with a low risk of NTD, defined as E-folate concentrations >906 nmol/L [23]. Additional studies are needed to characterise the women with E-folate concentration <906 nmol/L and to determine whether they would benefit from additional intakes of folic acid. One of the limitations of the present study is the lack of information on methylenetetrahydrofolate reductase (MTHFR), which is the rate-limiting enzyme in the pathway of one-carbon metabolism. Single nucleotide polymorphisms in MTHFR may influence susceptibility to NTD and other conditions that are associated with folic acid metabolism, and is important in determining E-folate concentrations in response to folic acid intake.

Only one participant in the present study had E-folate concentrations below the recommended 300 nmol/L. Folate fortification in Australia may have been successful in increasing folate status of young women and confirmation will be required from national representative data. Despite the benefits of eradicating folate deficiency there is some evidence in the literature that high folate status may have undesirable effects such as, neurotoxicity and decline in cognitive function and epigenetic programming in utero [46].

Mean concentrations of tHcy in the present study were similar [47,48] or lower than others in young women [49] or in a random population sample [50]. Small doses of folate have been shown to reduce tHcy in a young population [49] so the high blood folate levels observed in the present study may be protective of hyperhomocysteinaemia, which was found in 2% of women in this study, half of that in previous reports in young women [51].

Vitamin B_12_ concentrations in the present study were comparable [52] or lower than concentrations reported in vegans and omnivores [50,52,53]. We found 11.3% of participants with vitamin B_12_ concentrations <120 pmol/L, and 4.7% with vitamin B_12_ deficiency, defined as serum MMA concentrations >0.34 μmol/L. In a similar age group reported in NHANES 1999–2002 [54], ≤3% of the population were vitamin B_12_ deficient, and marginal deficiency was more common, at approximately 14%–16%. Australian data show that 4.4% of young adults were vitamin B_12_ deficient [55]. We found a significant trend for lower serum tHcy and MMA concentrations as quintiles of serum vitamin B_12_ increased. A study in older Australians found that 22.9% had vitamin B_12_ < 185 pmol/L [56] whereas in young women, we found this level to be nearly double (42.1%). The results of the present study suggest that a substantial proportion of young women are deficient in vitamin B_12_, potentially during early pregnancy, and the impact on maternal and foetal wellbeing should be considered.

Strengths of the present study include the availability of multiple analyses of micronutrient biomarkers, which allowed for assessing the prevalence of deficiency, and understanding the interrelationship of multiple deficiencies. Limitations of the present study are the lack of analysis of inflammatory markers since infection and low-grade inflammation may be confounders in the interpretation of iron [25], zinc [57] and other biomarkers of nutritional status [58]; the transitional period of folate fortification during the study period; and the potential sample bias due to the selection of volunteers who were prepared to cease taking nutritional supplements prior to their participation in the present study. Despite strict exclusion of participants with any illness, there is the possibility that acute inflammation may have been present.

## 5. Conclusions

Based on selected biomarker analyses in female students we observed both micronutrient deficiencies and excesses; including iron deficiency, vitamin B_12_ deficiency, and suboptimal selenium among the majority of females. In contrast, folate status was beyond the upper levels of the reference range for serum and erythrocyte folate concentrations. The extent of the health effects of multiple micronutrient deficiencies and excesses on health outcomes, including pregnancy outcomes in a healthy population is of concern. Further research is needed to address nutrient deficiencies in this population so effective public health interventions can be developed.
